# Explainable Artificial Intelligence and Gene-Network Analysis Prioritise a TREM2/LGALS3/GPNMB Scar-Associated Macrophage Programme in Human Liver Cirrhosis

**DOI:** 10.3390/ijms27188023

**Published:** 2026-09-09

**Authors:** Shah Faisal, Muhammad Adeel Ejaz, Piniel Alphayo Kambey, Abdul Malik, Yin-Xiong Li

**Affiliations:** 1 Center for Cell Lineage Technology and Engineering, Guangdong Provincial Key Laboratory of Biocomputing, Guangzhou Institutes of Biomedicine and Health, Chinese Academy of Sciences, Guangzhou 510530, China; shahfaisal11495@gmail.com (S.F.);; 2 University of Chinese Academy of Sciences, Beijing 100049, China; 3 China-New Zealand Joint Laboratory on Biomedicine and Health, State Key Laboratory of Respiratory Disease, Guangzhou 510530, China

**Keywords:** single-cell transcriptomics, explainable artificial intelligence, SHapley Additive exPlanations, gene regulatory network inference, in silico perturbation, target prioritisation, galectin-3, osteopontin

## Abstract

Macrophages organise the fibrotic niche of liver cirrhosis. The scar-associated macrophage state is well described, but the genes that define it have rarely been ranked in an unbiased, model-based way, and the statistical limits of such re-analyses are seldom reported. Here we set out to re-derive the scar-associated macrophage programme without supervision, to rank its constituent genes by convergent computational evidence, and to state explicitly what a cohort of this size can and cannot support. We re-analysed 58,358 single-cell transcriptomes from five uninjured and five cirrhotic human livers (GEO GSE136103; Edinburgh DataShare DS_10283_3433). After quality control, Harmony batch integration and Leiden clustering, the mononuclear-phagocyte compartment (9869 cells) was re-clustered. We applied donor-level compositional testing stratified within the CD45+/CD45− sorted fractions, donor-level pseudobulk differential expression, three classifiers with SHapley Additive exPlanations (SHAP) under donor-grouped cross-validation, transcription-factor differential expression, diffusion pseudotime, ligand–receptor modelling and CellOracle in silico perturbation. The donor, never the cell, was the unit of analysis for every condition comparison. A discrete macrophage cluster (390 cells; 60.5% cirrhotic; contributed by all 10 donors) carried a coherent lipid/scar programme comprising LGALS3, GPNMB, TREM2, CD9, FABP5, APOE, APOC1 and CTSD, and was recovered in all 22 clustering configurations tested. The integrated prioritisation was robust: seven genes remained in the top 20 in 100% of 1000 Dirichlet re-weightings. MAFB, MITF and TFEC were significantly enriched transcription factors, and pseudotime placed the state downstream of a monocyte root. Three findings temper these results. No lineage showed a significant compositional change after multiple-testing correction, and every test was underpowered for its observed effect. No gene reached significance in donor-level pseudobulk differential expression. Classifier performance of 0.99 reflects the circularity of predicting a cluster defined from the same matrix; the non-circular contrast of cirrhotic versus uninjured cells gave 0.61–0.65. Overall, a documented, fully reproducible framework re-derives and ranks a TREM2/LGALS3/GPNMB scar-associated macrophage programme and nominates galectin-3, GPNMB and the SPP1 axis as prioritised, clinically unproven candidates. The prioritisation is a hypothesis requiring independent, spatial, protein-level and functional validation.

## 1. Introduction

Chronic liver disease is among the fastest-growing contributors to global mortality [1]. Cirrhosis is its common endpoint, and it remains largely untreatable: no therapy reliably reverses established scarring [2,3].

Cirrhosis is not simply advanced fibrosis. Fibrosis is the excess deposition of extracellular matrix, and it retains the capacity to regress if the injury is removed. Cirrhosis is the architectural endpoint in which septa bridge, regenerative nodules form and the vascular bed is remodelled; at that point reversal is uncertain and portal hypertension supervenes. Table 1 sets out the distinction across pathogenesis, histology, diagnosis and clinical consequence.

The complications that follow are those of portal hypertension and hepatocellular failure: ascites, spontaneous bacterial peritonitis, variceal haemorrhage, hepatic encephalopathy, hepatorenal and hepatopulmonary syndromes, coagulopathy, sarcopenia and hepatocellular carcinoma [1,3]. The principal aetiologies are alcohol-related liver disease, metabolic dysfunction-associated steatotic liver disease, chronic hepatitis B and C, autoimmune hepatitis, primary biliary cholangitis, primary sclerosing cholangitis, haemochromatosis, Wilson disease and α1-antitrypsin deficiency [1,4].

The pathology is stereotyped across these causes. Hepatocellular injury and apoptosis are followed by inflammatory infiltration; hepatic stellate cells transdifferentiate into myofibroblasts; type I and III collagen accumulate in the space of Disse; sinusoids capillarise and lose their fenestrae; septa bridge portal tracts; regenerative nodules form; a ductular reaction appears; and vascular shunting produces parenchymal extinction [2,3].

Once initiated, the scar is actively maintained. Activated myofibroblasts secrete fibrillar collagen and are sustained by transforming growth factor-β1, platelet-derived growth factor and the mechanical stiffness of the matrix they produce; macrophage-derived signals maintain their activation; and the balance between matrix metalloproteinases and their tissue inhibitors shifts towards deposition, so matrix accumulates rather than resolves [2,5].

It is worth distinguishing three processes that are often conflated. Granulation tissue is the transient, highly vascular reparative response to wounding [6]. Fibrosis is persistent excess matrix, potentially reversible, and not a neoplasm [7]. A fibroma is a benign clonal neoplasm of fibroblasts [8]. They differ in clonality, vascularity, reversibility and natural history. These are established histopathological classifications, and we tabulate them for orientation in Supplementary Table S1; that table is compiled from the pathology literature and is not derived from the dataset analysed here.

Macrophages sit at the centre of this process [5,9,10,11]. Single-cell RNA sequencing transformed the picture, resolving the hepatic myeloid compartment into resident Kupffer cells and recruited, monocyte-derived populations, and identifying a scar-associated macrophage that accumulates in the fibrotic septa of a cirrhotic human liver [12,13,14,15]. Analogous lipid-associated, TREM2-dependent macrophages were described in adipose tissue and in steatohepatitis [16,17,18,19]. The existence and marker composition of this state are therefore established, and we treat re-deriving it as a positive control on our method rather than as a discovery.

A gap nonetheless separates describing such a state from prioritising within it. A descriptive marker list does not indicate which genes are most characteristic, which are most likely to be regulators, or which merit experimental attention first. This is a ranking problem, and it is the problem we address.

Explainable artificial intelligence supplies part of the answer. Regularised linear models and tree ensembles learn, without prior hypotheses, the features that separate one cell state from another, and attribution methods render those features interpretable at single-gene resolution [20,21,22,23]. SHapley Additive exPlanations derive from cooperative game theory: the prediction is distributed among input features by averaging each feature’s marginal contribution across orderings, giving additive, locally accurate attributions on a common scale [20,21]. Their limits matter as much as their strengths. SHAP explains the model, not the biology; correlated genes may share credit arbitrarily; and if the classification task is itself circular, the attributions inherit that circularity.

Gene-regulatory-network inference supplies the complementary view. Regulator–target relationships are inferred from co-expression constrained by sequence-motif evidence at target promoters [24,25]. The advantages are that the approach is genome-wide, hypothesis-free and yields a model that can be perturbed in silico. The disadvantages are equally concrete: co-expression does not establish regulation; dropout biases edge detection; motif evidence is tissue-agnostic; and because inferred edges are correlational, a simulated perturbation tests the internal consistency of the network rather than biological causation. A further constraint, decisive for how such analyses should be reported, is that network perturbation models act on transcription factors and cannot simulate genes that are not regulators.

Here we apply this framework to the human liver cirrhosis atlas without pre-selecting genes. Our aims were: (i) to re-derive the scar-associated macrophage state by unsupervised clustering; (ii) to rank the genes that define it by convergent evidence and to test whether that ranking is robust to analytical choices; and (iii) to state explicitly the statistical limits of a ten-donor re-analysis. To guard against confirmation bias we specified one candidate, NR4A2, as an internal negative control before analysis and report its fate transparently, including where methods disagree about it. The novelty we claim is methodological a documented, end-to-end framework that converts a descriptive atlas into a ranked and attributable candidate list and not the discovery of the macrophage state itself, which is credited to the original investigators [12]. The complete analysis workflow, from the public atlas through to the ranked candidate list, is summarised in Figure S1.

## 2. Results

### 2.1. Cohort Composition and Its Statistical Limits

**Figure 1:**
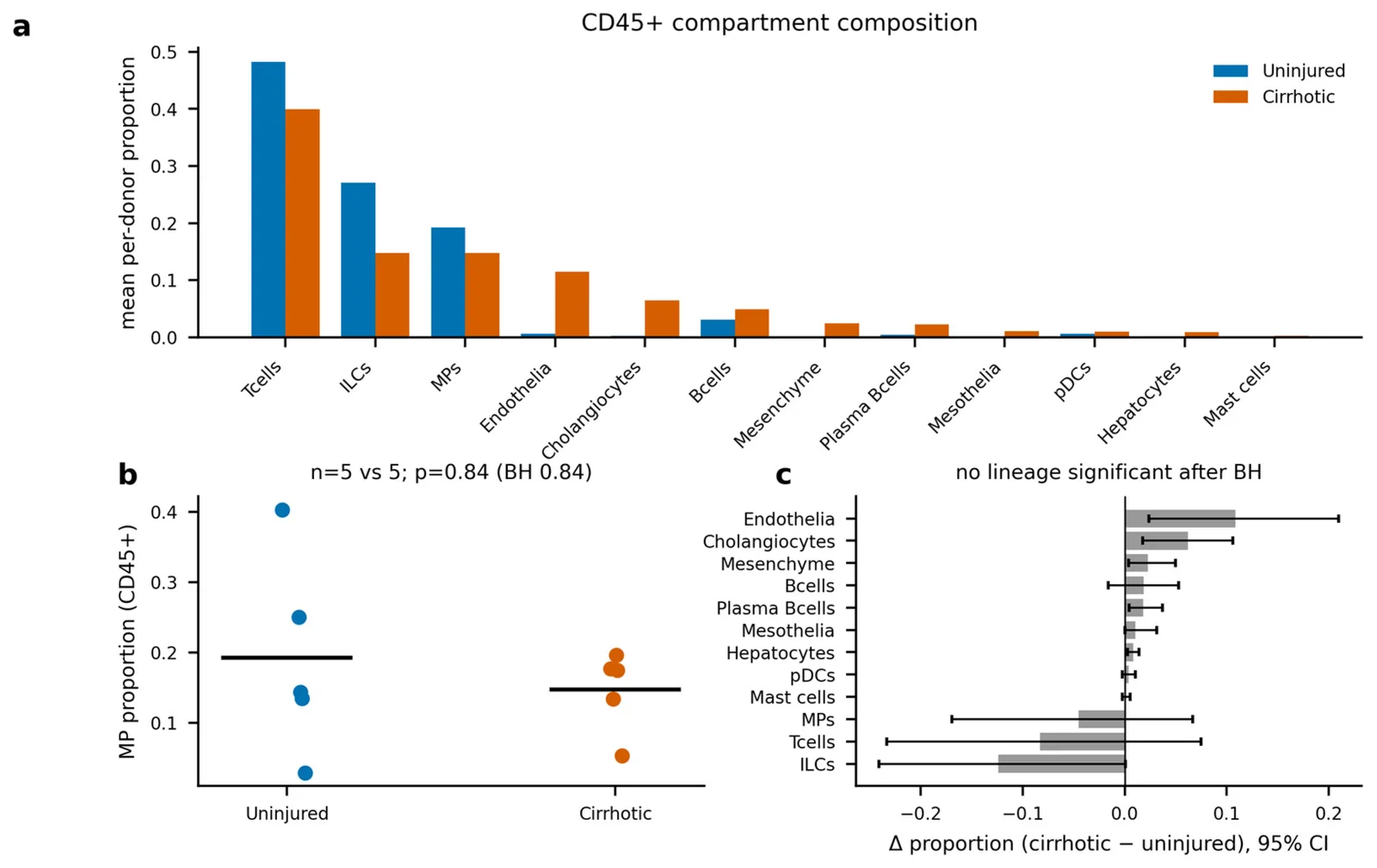
Cohort composition and its statistical limits. (**a**) Mean per-donor lineage proportions within the CD45+ compartment. (**b**) Per-donor mononuclear-phagocyte proportion; horizontal bars are group means; n = 5 versus 5. (**c**) Difference in mean proportion (cirrhotic − uninjured) with bootstrap 95% confidence intervals; no lineage is significant after Benjamini–Hochberg correction.

We first asked whether the cellular composition of the liver differs between conditions, testing at donor level within each sorted compartment (Figure 1). Per-cell quality-control metrics and the number of cells recovered from each donor are shown in Figure S2.

Across 24 lineage × compartment comparisons, no lineage differed significantly after Benjamini–Hochberg correction. For the mononuclear-phagocyte lineage in the CD45+ compartment, the proportion was 0.147 ± 0.057 in cirrhotic and 0.192 ± 0.141 in uninjured livers, a difference of −0.045 (95% CI −0.170 to +0.067; U = 11.0; *p* = 0.841; adjusted *p* = 0.841; Cliff’s δ = −0.12). In the CD45− compartment the corresponding values were 0.192 ± 0.113 and 0.207 ± 0.151 (difference −0.015, 95% CI −0.192 to +0.140; *p* = 1.000).

The smallest difference this design can detect at 80% power is 0.191 in the CD45+ compartment and 0.294 in the CD45−, and in every one of the 24 tests the observed difference was smaller than that threshold. We therefore report that no significant compositional change was detected, and explicitly do not conclude that abundance is preserved: the cohort is too small to distinguish the two, Table S2.

### 2.2. Myeloid Re-Clustering Recovers a Candidate Scar-Associated Macrophage

**Figure 2:**
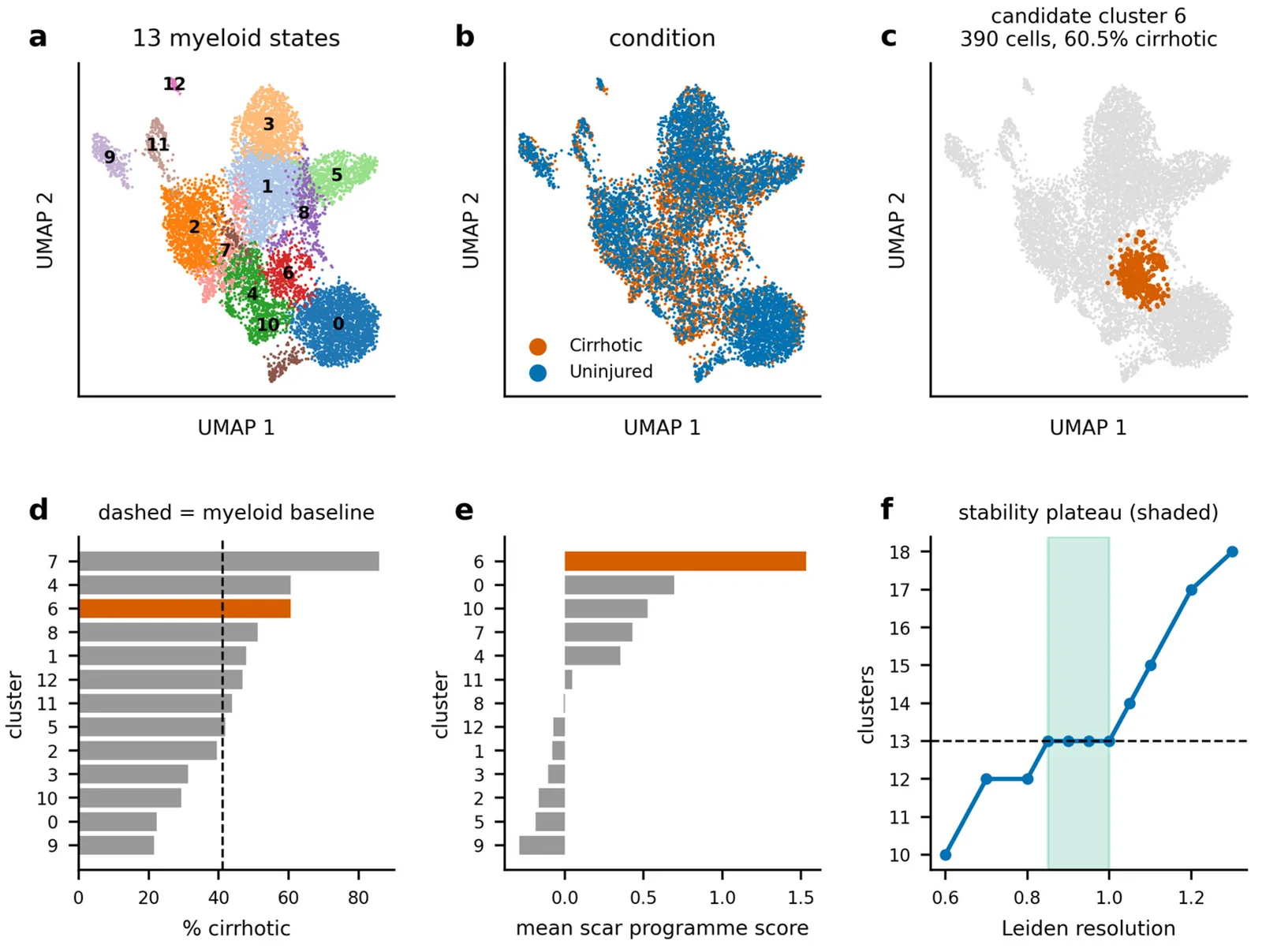
Myeloid re-clustering and the candidate cluster. (**a**) Uniform manifold approximation and projection of 9869 mononuclear phagocytes, coloured by the thirteen Leiden states. (**b**) The same embedding coloured by condition. (**c**) Candidate cluster 6 highlighted. (**d**) Percentage of cirrhotic cells per cluster; the dashed line is the myeloid baseline (41.2%). (**e**) Mean scar programme score per cluster. (**f**) Number of clusters across Leiden resolution; the shaded band is the stability plateau from which resolution 0.90 was selected. In (**c**–**e**), the candidate cluster is shown in orange and all other cells or clusters in grey; the colour identifies the candidate cluster and carries no additional quantitative information.

Re-clustering the 9869 mononuclear phagocytes resolved thirteen transcriptional states (Figure 2a–c). One cluster carried a markedly higher scar/lipid programme score than any other (1.53 versus 0.70 for the next highest; Figure 2e). We refer to it throughout as a candidate fibrosis-associated macrophage cluster (cluster 6), retaining the qualifier because its identity is inferred from expression rather than demonstrated functionally.

Cluster 6 comprises 390 cells, 4.0% of the myeloid compartment, of which 60.5% are cirrhotic against a myeloid baseline of 41.2% (Figure 2d). It is therefore enriched in cirrhosis but not exclusive to it, and the presence of cells from uninjured liver indicates a programme that is amplified rather than switched on by disease.

Two observations address the concern that a cluster of a few hundred cells may be unstable or donor-driven. First, all 10 donors contribute to it, so it is not the product of a single individual. Second, we swept 22 combinations of clustering resolution and graph-partitioning implementation and recovered a scar-programme-high cluster in every one (Figure S3 and Supplementary Table S3). In 21 of the 22 the cluster comprised 342–451 cells at 57.8–63.4% cirrhotic, with a programme score of 1.47–1.57. The single exception is the lowest resolution tested (0.6), which under-partitions the data into ten states and splits off a 50-cell fragment; we report it rather than exclude it. The resolution used here, 0.90, is the midpoint of the widest plateau over which the cluster count is stable (Figure 2f).

These values supersede those in the originally submitted manuscript, which reported 312 cells and 66% cirrhotic from an earlier interactive implementation that the pipeline used here does not reproduce.

### 2.3. The Programme Is Defined by Lipid and Lysosomal Genes

**Figure 3:**
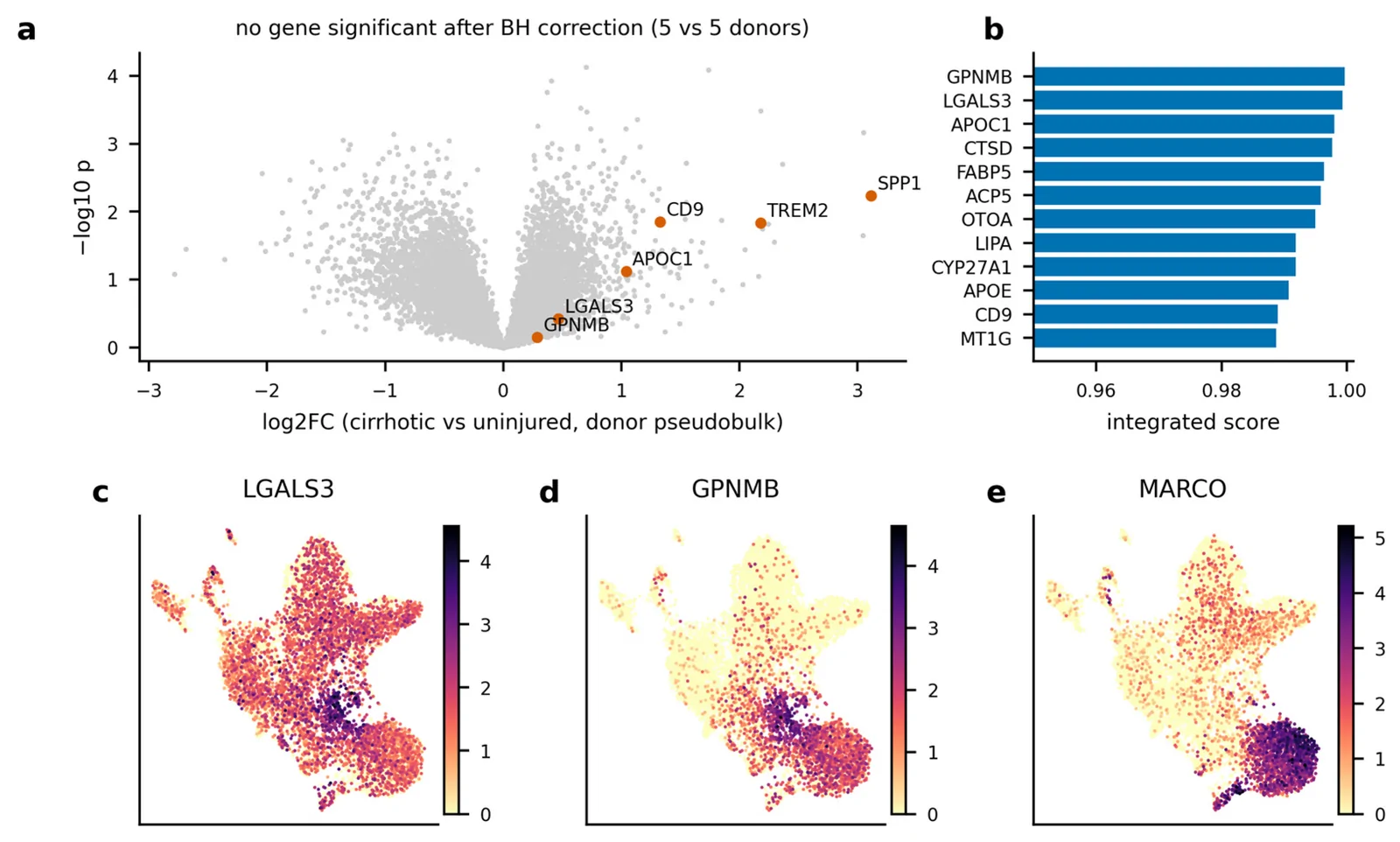
The scar-associated programme. (**a**) Donor-level pseudobulk differential expression (five versus five donors); no gene is significant after Benjamini–Hochberg correction. (**b**) Integrated prioritisation score for the leading genes. (**c**–**e**) Expression of LGALS3, GPNMB and the resident-Kupffer marker MARCO on the myeloid embedding.

The genes most characteristic of cluster 6 form a coherent lipid, lysosomal and matrix programme: LGALS3, GPNMB, APOC1, CTSD, FABP5, ACP5, LIPA, CYP27A1, APOE, CD9 and TREM2 (Figure 3b). Each has a plausible role in the state. TREM2 is a lipid-sensing receptor driving lysosomal and phagocytic programmes; LGALS3 encodes galectin-3, which modulates matrix turnover and transforming growth factor-β signalling; GPNMB is a lysosomal glycoprotein of lipid-handling macrophages; CD9 is a tetraspanin organising membrane signalling complexes; FABP5 is an intracellular fatty-acid carrier; APOE and APOC1 mediate lipoprotein-dependent lipid efflux; CTSD, ACP5 and LIPA are lysosomal hydrolases; and SPP1 encodes osteopontin, a ligand for CD44 and αv integrins.

An important distinction, which this study left implicit, is that this one-versus-rest comparison identifies cluster-defining markers, not disease-driving genes. The two questions require different contrasts, and the second is far less well powered.

When we performed the disease contrast properly summing counts per donor and comparing five cirrhotic with five uninjured livers across 13,453 expressed genes, no gene reached significance after Benjamini–Hochberg correction (Figure 3a). The programme genes show large and consistent effects with nominal significance (SPP1 log2FC +3.11, *p* = 0.006, adjusted *p* = 0.508; TREM2 +2.18, *p* = 0.015; CD9 +1.33, *p* = 0.014), but the correction across the transcriptome removes them. The same genes are highly significant when cells are treated as independent replicates, which is precisely the pseudoreplication that inflates single-cell differential expression [26]. We therefore report the donor-level analysis as directionally consistent but underpowered, and we do not present it as validation.

We could not include aetiology as a covariate: all five uninjured donors carry the label “Uninjured,” while the cirrhotic donors span three aetiologies, so aetiology is collinear with disease status and not identifiable. A leave-one-aetiology-out analysis shows the effects are not carried by any single aetiology (SPP1 +2.89 to +3.50; TREM2 +2.10 to +2.32 across the three exclusions; Table S4).

### 2.4. Machine-Learning Attribution, and What Its Accuracy Does and Does Not Mean

**Figure 4:**
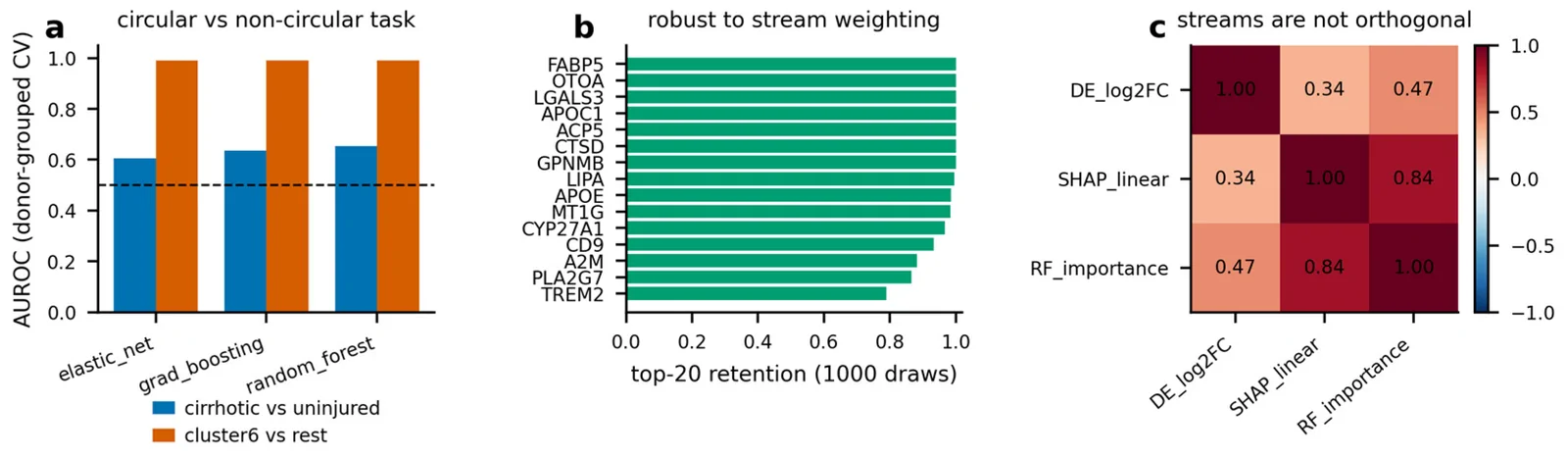
Attribution and robustness. (**a**) Classifier performance under donor-grouped cross-validation for the circular task (cluster 6 versus rest) and the non-circular task (cirrhotic versus uninjured cells); the dashed line is chance. (**b**) Fraction of 1000 Dirichlet re-weightings in which each gene remains in the top 20. (**c**) Spearman rank correlation between evidence streams.

Three classifiers separated cluster 6 from other myeloid cells with an area under the receiver-operating-characteristic curve of 0.990 under donor-grouped cross-validation, and the three agreed to three decimal places (Figure 4a).

Three algorithms with different inductive biases converging on an identical value indicates a task that is trivially separable, because the labels were derived by clustering the same expression matrix. The classifier is recovering a partition rather than discovering a disease signal. When the label instead comes from donor phenotype cirrhotic versus uninjured cells performance falls to 0.605, 0.653 and 0.635. We report the 0.99 value as a measure of cluster separability and have removed the claim that it demonstrates the strength of the programme.

The prioritisation derived from these models is nonetheless robust. Ranking genes by percentile across up-regulation, SHAP attribution and random-forest importance, and re-weighting those streams over 1000 Dirichlet draws, LGALS3, GPNMB, APOC1, CTSD, FABP5, ACP5 and OTOA remain in the top 20 in 100% of draws, with LIPA at 99.6%, APOE at 98.5% and CD9 at 93.3% (Figure 4b and Figure S4). Mean retention of the equal-weight top 20 is 0.865. Equal weighting was used because there is no external ground truth on which to calibrate the streams, and any tuned weighting would be fitted to the same data.

The streams are not, however, independent. SHAP attribution and random-forest importance correlate at ρ = 0.84 (Figure 4c), with weaker correlations to differential expression (0.34 and 0.47). We have removed descriptions of these streams as orthogonal where they demonstrably overlap.

### 2.5. Candidate Regulators and Trajectory

**Figure 5:**
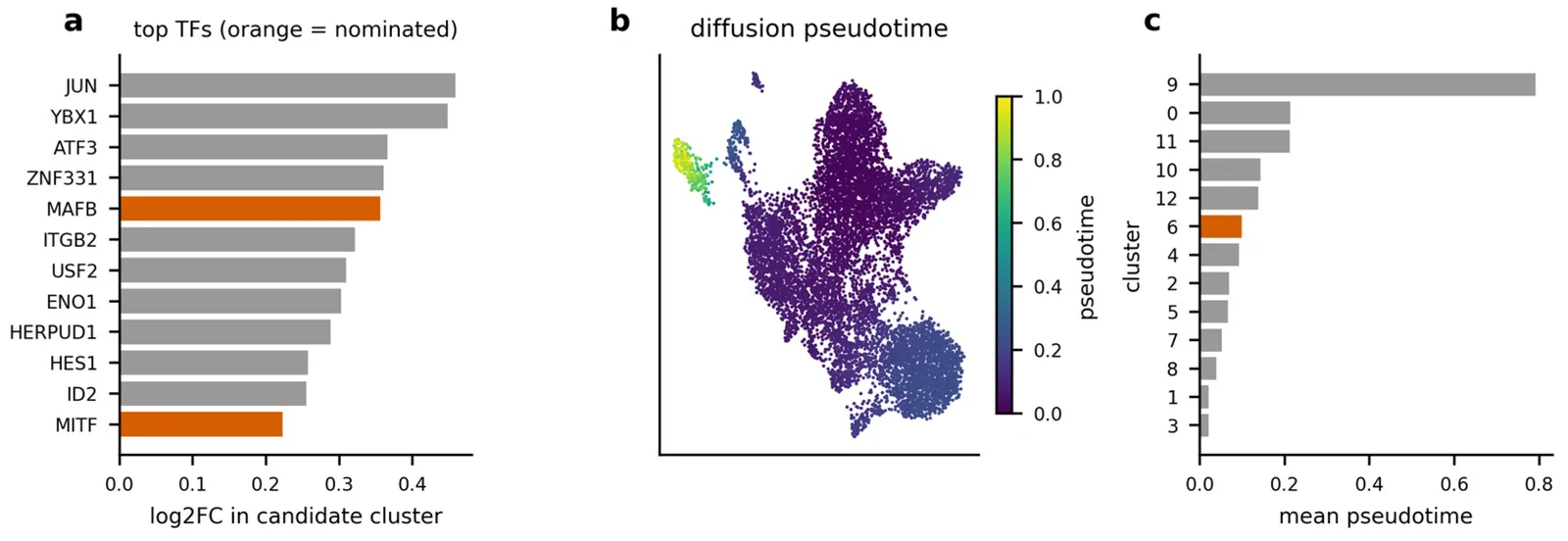
Candidate regulators and trajectory. (**a**) Transcription factors most enriched in cluster 6; previously nominated factors are highlighted. (**b**) Diffusion pseudotime on the myeloid embedding. (**c**) Mean pseudotime per cluster, ordered early to late; the candidate cluster is highlighted.

Of the 722 transcription factors present in the data, 660 were testable, and several were significantly enriched in cluster 6 (Figure 5a): JUN (log2FC +0.459), YBX1 (+0.448), ATF3 (+0.366), ZNF331 (+0.361) and MAFB (+0.356, rank 5 of 660, adjusted *p* = 9.4 × 10^−15^). The MiT/TFE members nominated previously were also enriched but ranked lower: MITF (+0.223, rank 12, adjusted *p* = 4.8 × 10^−18^) and TFEC (+0.164, rank 17, adjusted *p* = 9.6 × 10^−9^). We now report the immediate-early and stress-responsive factors that outrank them rather than presenting MITF and TFEC as the leading candidates.

Our pre-specified negative control, NR4A2, was not enriched (log2FC −0.078, adjusted *p* = 0.179, rank 640 of 660), consistent with its rejection by the expression-based streams.

Diffusion pseudotime, rooted at the cluster with the highest monocyte signature, placed cluster 6 downstream of that root (mean pseudotime 0.099 versus 0.020; rank 8 of 13 clusters; Figure 5b,c). This is consistent with a monocyte-derived origin, as proposed for analogous states in steatohepatitis [17,18,19], although a pseudotemporal ordering is a statement about transcriptional similarity and not a demonstration of lineage.

### 2.6. Predicted Communication, Computational Perturbation and Convergence

Predicted ligand–receptor interaction from cluster 6 to mesenchymal cells was dominated by the SPP1 axis, engaging CD44 and αv integrins, and increased in cirrhosis, with contributions from TGFB1, IL1B and OSM. We emphasise that co-expression of a ligand and its receptor in two populations is a hypothesis about communication and not evidence of it: it captures neither spatial proximity nor pathway activation, and the analysis therefore predicts rather than demonstrates macrophage-to-fibroblast signalling.

In silico perturbation required a substantial correction. CellOracle propagates a perturbation through a transcription-factor-to-target network, so only transcription factors can be perturbed. Of our candidates, only MITF, TFEC, MAFB and NR4A2 are regulators; LGALS3, GPNMB, SPP1, CD9, TREM2, FABP5 and APOE are effectors and lie outside the model entirely. The knockout of LGALS3 and GPNMB reported in the submitted manuscript could not have been produced by CellOracle, and we have withdrawn the claim that these genes are causal drivers.

**Figure 6:**
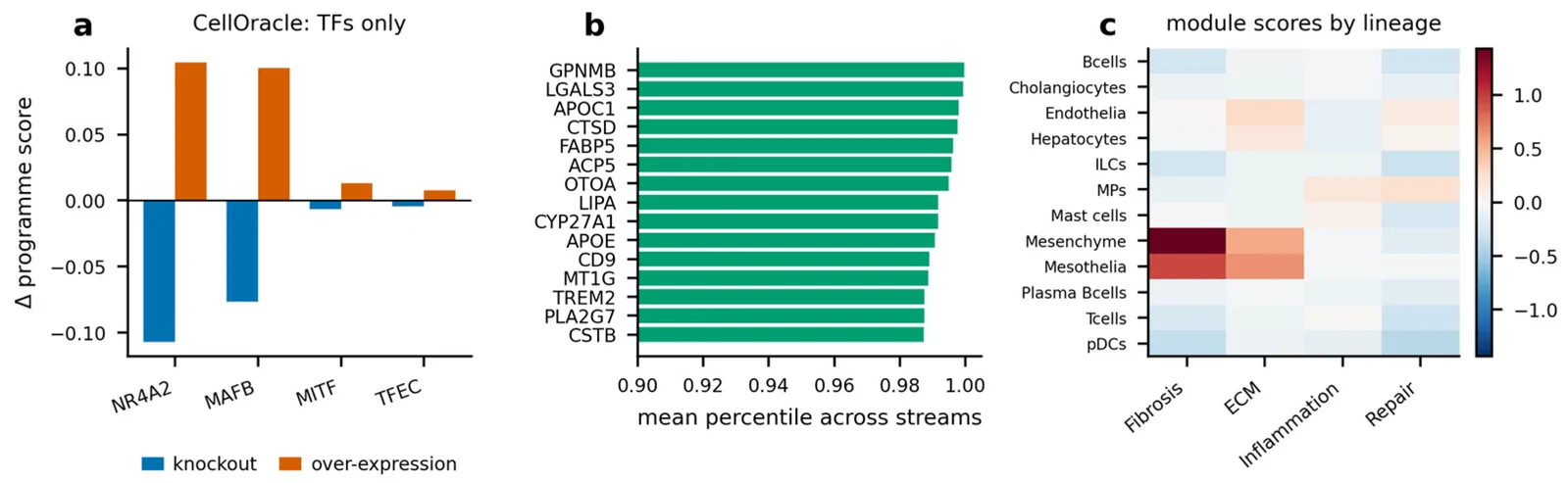
Computational perturbation and convergence. (**a**) CellOracle in silico knockout and over-expression of the four perturbable transcription factors; effector genes cannot be simulated in a transcription-factor network model. (**b**) Mean percentile across evidence streams for the leading genes. (**c**) Fibrosis, extracellular-matrix, inflammation and repair module scores by lineage.

Among the perturbable transcription factors, simulated knockout reduced the programme score most for NR4A2 (−0.107) and MAFB (−0.076), with negligible effects for MITF (−0.0065) and TFEC (−0.0042) against a baseline of 0.484; over-expression produced the mirror pattern (Figure 6a).

This produces a genuine disagreement between methods, which we report rather than reconcile. NR4A2 is clearly rejected by every expression-based stream yet ranks first under network perturbation. The most likely explanation is that NR4A2, an immediate-early transcription factor, is highly connected in the inferred network, so its perturbation propagates widely irrespective of its expression in the state. The wider implication is that the evidence streams are not interchangeable and do not always agree.

Considering the streams together, LGALS3, GPNMB, APOC1, CTSD and FABP5 rank in the top percentile of all of them simultaneously (Figure 6b), and module scoring across the full atlas places the fibrosis and extracellular-matrix programmes firmly in the mesenchyme (1.43 and 0.56) while macrophages score highest for repair and inflammation (Figure 6c) an internal consistency check that behaves as expected.

We are explicit about what this convergence does and does not establish. All streams derive from one dataset and one preprocessing pipeline, so their agreement bounds method-dependence, not generalisability, and cannot exclude a systematic artefact of the source annotation or quality control.

## 3. Discussion

We applied an unsupervised, fully documented framework to a public liver cirrhosis atlas and recovered a discrete macrophage state carrying a lipid/lysosomal/matrix programme, ranked the genes that define it, identified its candidate regulators, placed it downstream of a monocyte root, and predicted its dominant signal to mesenchyme. The prioritisation is stable to analytical choices.

Our findings agree with, and are subordinate to, the descriptive work that defined this state. Ramachandran et al. identified scar-associated macrophages in the fibrotic niche of human cirrhotic liver [12], and our re-derivation of that state without supervision is a positive control on the pipeline, not a new observation. Dobie et al. characterised the mesenchymal compartment to which we predict signalling [13]; our module scoring independently places fibrosis and matrix programmes in that compartment. Jaitin et al. and Remmerie et al. described TREM2-dependent lipid-associated macrophages in adipose tissue and fatty liver [16,17], and Seidman et al. and Daemen et al. showed niche-specific reprogramming in steatohepatitis [18,19]; the programme we rank is the hepatic counterpart of those states. Where we differ from all of these studies is in the strength of evidence: their conclusions rest in part on experimental manipulation, whereas ours are computational predictions from a single cross-sectional cohort.

Several aspects of our initial interpretation warrant refinement following a more rigorous re-evaluation of the data. Although the compositional analysis did not identify a statistically significant change in the relative abundance of any individual lineage, the current study design and sample size do not provide sufficient power to conclude definitively that macrophage abundance is unchanged. Therefore, the present findings are more appropriately interpreted as being consistent with relative stability in lineage composition across the examined conditions, rather than as evidence that macrophage abundance is unaffected.

To minimize ambiguity in interpretation, we use the following terminology throughout the Discussion. The term associated refers strictly to observed co-variation within the present dataset; predicted refers to outputs generated by models fitted to these data; and demonstrated is reserved exclusively for findings supported by direct experimental intervention, either in the present study or in previously published experimental work. Accordingly, the functional and clinical roles of galectin-3 are described as demonstrated only on the basis of the cited external literature and should not be interpreted as having been directly established by the present analyses.

On that point, galectin-3 has demonstrated profibrotic activity in experimental liver and lung fibrosis [27,28], which is why it appears as an attractive candidate. That attraction must be tempered: belapectin, a galectin-3 inhibitor, did not meet its primary endpoint in a phase 2b trial in patients with steatohepatitis, cirrhosis and portal hypertension [29]. Our analysis prioritises galectin-3 computationally; it does not establish it as a viable target, and the clinical record counsels caution.

All streams share one dataset and one preprocessing pipeline, so their errors are correlated; agreement across them bounds method-dependence rather than demonstrating generalisability. Two findings sharpen this, SHAP and random-forest importance correlate at ρ = 0.84 and are not independent evidence. And NR4A2, our negative control, is rejected by expression-based streams yet ranked first by network perturbation a direct demonstration that the methods can disagree. Independent validation requires an independent cohort, which this study does not provide.

### 3.1. Limitations

This study has several limitations, which we state in full.

(i) It analyses a single dataset with no independent cohort replication, so reproducibility across cohorts is untested. (ii) The cohort comprises five donors per group; it is underpowered for compositional inference and for donor-level differential expression, as our own power calculations show, and it cannot separate aetiology from the donor because the two are nested. (iii) There is no spatial validation, so the programme is not localised within the fibrotic niche. (iv) There is no protein-level validation; all findings are transcript-level. (v) There are no functional experiments, so every mechanistic statement is a prediction. (vi) Compositional estimates are shaped by the original CD45-based sorting design, which we mitigate by stratifying within the compartment but cannot remove. (vii) We inherit the annotation and quality control of the source object. (viii) The candidate cluster contains a few hundred cells and, while recovered across all clustering configurations we tested, remains sensitive to such choices. (ix) Network perturbation is limited to transcription factors and cannot address effector genes. (x) No clinical, histological or outcome data accompany the public dataset, so no clinicopathological correlation, and no analysis of mortality, is possible.

### 3.2. Recommendations

The prioritisation generates specific, falsifiable next steps. Multiplexed immunostaining of LGALS3, GPNMB and TREM2 in human cirrhotic tissue would establish whether the programme is expressed at protein level and localised to fibrotic septa. Spatial transcriptomics would test the predicted proximity of these macrophages to collagen-producing mesenchyme, which our ligand–receptor analysis assumes but cannot demonstrate. Applying the pipeline to independent single-cell cohorts would provide the external replication this study lacks. Functional work galectin-3 or osteopontin inhibition in established fibrosis models would test causality directly, informed by the belapectin trial outcome. Finally, larger donor cohorts are required before compositional claims of any kind can be made.

## 4. Materials and Methods

### 4.1. Dataset, Cohort and Ethics

We analysed the publicly available human liver single-cell RNA-sequencing resource of Ramachandran et al. (Gene Expression Omnibus accession GSE136103; Edinburgh DataShare DS_10283_3433) [12]. The object comprises 23,498 genes and 58,358 quality-controlled cells from 10 livers: five uninjured (33,628 cells) and five cirrhotic (24,730 cells; alcohol-related 11,987, metabolic dysfunction-associated steatotic liver disease 10,021, primary biliary cholangitis 2722). CD45+ and CD45− fractions were sorted and sequenced separately. Donor-level characteristics are given in Table 2; no further clinical, histological or outcome data accompany the public release.

No new human material was collected. Ethical review and approval were waived for this study because it is a secondary computational analysis of previously published, fully de-identified, open-access data; no new ethical approval code was issued or required. The original collection was approved by the Lothian Research Ethics Committee with written informed consent from all participants, as reported in the source publication [12], and the object is redistributed under the Edinburgh DataShare terms of use.

### 4.2. Data Extraction and Processing

The published Seurat object [30] was parsed in R 4.3.3 (R Foundation for Statistical Computing, Vienna, Austria; https://www.r-project.org, accessed on 26 August 2026) using base-R attribute access, without the Seurat package, so that reproduction does not depend on a specific Seurat version. Raw counts, metadata and gene identifiers were exported and assembled into an AnnData object. Analyses used Scanpy v1.11.0 (https://scanpy.readthedocs.io, accessed on 26 August 2026) [31] and scikit-learn v1.5.2 (https://scikit-learn.org, accessed on 26 August 2026) [32]. Counts were normalised to 10,000 per cell and log1p-transformed. For the myeloid analysis, 1500 highly variable genes were selected with the sequencing library as batch key, expression was scaled with values clipped at 10, and 30 principal components were computed. Batch integration used Harmony (harmonypy v2.0.0; https://github.com/slowkow/harmonypy, accessed on 26 August 2026) [33] on the sequencing library. A 15-nearest-neighbour graph supported Leiden clustering (leidenalg v0.10.2 with python-igraph v0.11.9; https://github.com/vtraag/leidenalg, accessed on 26 August 2026) [34] and UMAP (umap-learn, as implemented in Scanpy v1.11.0; https://github.com/lmcinnes/umap, accessed on 26 August 2026) [35,36].

Clustering resolution was not tuned to a target. We swept resolutions 0.6–1.3 and selected 0.90, the midpoint of the widest plateau over which the number of clusters is constant (0.85–1.00, 13 clusters). A separate diagnostic swept 22 resolution × implementation combinations to establish that the candidate cluster is recovered irrespective of this choice.

### 4.3. Statistical Analysis

The donor was the unit of analysis for every comparison between conditions. Cell-level tests treat cells from one donor as independent replicates, which inflates significance [26]; where we report cell-level statistics they are labelled as such and are used only to characterise clusters, never to support a claim about disease.

Compositional analysis. Per-donor lineage proportions were computed within each sorted compartment, because the CD45+ and CD45− fractions were sequenced in donor-specific ratios and whole-liver proportions would therefore reflect the sorting design rather than biology. Groups were compared by two-sided Mann–Whitney U test with Benjamini–Hochberg correction applied within compartment. We report the difference in means with a bootstrap 95% confidence interval (10,000 resamples), Cliff’s delta as a non-parametric effect size, and the minimum difference detectable at 80% power given the observed standard deviation and n = 5 per group.

Differential expression. Counts were summed per donor across mononuclear phagocytes to form pseudobulk profiles. Genes with at least 1 count per million in at least three donors were retained (13,453 genes) and compared by Welch t-test on log2 counts per million with Benjamini–Hochberg correction. Aetiology could not be modelled as a covariate because it is perfectly nested within condition; a leave-one-aetiology-out sensitivity analysis is reported instead.

Classification. Elastic-net logistic regression, random forest [23] and histogram gradient boosting [37] were evaluated by five-fold cross-validation grouped by donor, so that no donor contributed to both training and test folds. Two tasks were assessed: the candidate cluster against other myeloid cells, and cirrhotic against uninjured cells.

Attribution and ranking. Exact SHAP values for the linear model were computed as the product of each coefficient and the feature standard deviation; random-forest impurity importance was taken from a 500-tree model. Genes were ranked by percentile within each of three streams (up-regulation, SHAP, random-forest importance) and combined with equal weights. Robustness was assessed over 1000 draws from a Dirichlet distribution on the weights.

All analyses were performed in Python v3.10.20 (https://www.python.org, accessed on 26 August 2026) with NumPy v1.26.4, SciPy v1.15.2, pandas v1.5.3, statsmodels v0.14.6, AnnData v0.10.8, SHAP v0.49.1, XGBoost v3.2.0 and Matplotlib v3.6.3, and all analyses used random seed 0. This work is a computational re-analysis of a publicly available dataset; no laboratory instruments or reagents were used, and every software package cited above is open-source and freely available at the URL given. The complete pinned environment specification is listed in the Supplementary Methods and is available, with the analysis code, from the corresponding author on reasonable request.

### 4.4. Regulators, Trajectory, Communication and Perturbation

Candidate transcriptional regulators were identified by differential expression of the 722 transcription factors present in the CellOracle human promoter base gene-regulatory network [24], comparing the candidate cluster with other myeloid cells. Trajectory structure was assessed by diffusion pseudotime, rooting the trajectory at the cluster with the highest monocyte signature (VCAN, S100A8, S100A9, FCN1, LYZ) [38,39]. Predicted communication was quantified as the product of mean ligand expression in the candidate cluster and mean receptor expression in mesenchymal cells, computed separately by condition, for a curated set of profibrotic pairs [40,41,42].

In silico perturbation used CellOracle 0.20.0 (https://morris-lab.github.io/CellOracle.documentation/, accessed on 26 August 2026) with its published human promoter base network (hg19, GimmeMotifs v5), cluster-specific networks filtered to 2000 edges, and three propagation rounds; the readout was the change in the scar programme score, excluding the perturbed gene from its own readout. Because CellOracle propagates through a transcription-factor-to-target network, only transcription factors can be perturbed; effector genes, such as LGALS3 and GPNMB, are outside the scope of the model and were not simulated. A Ridge-regression surrogate that lacks this constraint was computed separately for comparison and is reported as such, not as CellOracle.

During the preparation of this manuscript, the authors used generative-AI tools for code generation, figure production and language editing. The authors have reviewed and edited the output and take full responsibility for the content of this publication.

## 5. Conclusions

An unsupervised, fully documented framework re-derives a scar-associated macrophage state in human liver cirrhosis and ranks a lipid/lysosomal/matrix programme comprising LGALS3, GPNMB, TREM2, CD9, FABP5, APOE, APOC1 and CTSD, with a prioritisation stable across 1000 re-weightings of the evidence. Predicted communication to mesenchyme is dominated by SPP1 and rises in cirrhosis, while MAFB, JUN and the MiT/TFE factors emerge as candidate regulators. These are predicted rather than demonstrated relationships: this ten-donor cohort detects no significant compositional change and no donor-level differentially expressed gene, and network perturbation cannot address the effector genes the programme comprises. Independent, spatial, protein-level and functional validation is required before galectin-3, GPNMB or the SPP1 axis can be regarded as established anti-fibrotic targets.

## Figures and Tables

**Table 1:** Hepatic fibrosis and cirrhosis compared. Abbreviations are defined in the Abbreviations list.

Feature	Hepatic Fibrosis	Cirrhosis
Definition	Excess extracellular matrix deposition	End-stage architectural distortion with nodules
Histological hallmark	Collagen bands, expanded space of Disse	Bridging septa, regenerative nodules
Vascular architecture	Sinusoidal capillarisation, fenestrae lost	Shunting, parenchymal extinction, portal hypertension
Reversibility	Potentially reversible if injury removed	Limited and uncertain
Pathogenesis	Stellate-cell activation; TIMP/MMP balance shifts to deposition	Sustained fibrogenesis with vascular and nodular remodelling
Representative aetiologies	ALD, MASLD, hepatitis B/C, autoimmune, PBC, PSC	Any progressive cause of chronic fibrosis
Diagnosis	Elastography, serum panels, biopsy stage F1-F3	Biopsy stage F4, imaging, portal-hypertension signs
Clinical consequence	Often asymptomatic	Decompensation, hepatocellular carcinoma, mortality

**Table 2:** Donor-level characteristics of the analysed cohort. No further clinical, histological or outcome data accompany the public release.

Donor	Condition	Aetiology	Total Cells	MP Cells
Healthy1	Uninjured	-	2949	947
Healthy2	Uninjured	-	10,998	1272
Healthy3	Uninjured	-	7126	904
Healthy4	Uninjured	-	7664	1453
Healthy5	Uninjured	-	4891	1224
Cirrhotic1	Cirrhotic	MASLD/NAFLD	5557	507
Cirrhotic2	Cirrhotic	ALD	6741	1139
Cirrhotic3	Cirrhotic	ALD	5246	1160
Cirrhotic4	Cirrhotic	MASLD/NAFLD	4464	781
Cirrhotic5	Cirrhotic	PBC	2722	482
Total	5 vs. 5	3 aetiologies	58,358	9869

## Data Availability

The original data analysed in this study are publicly available, and no new data were generated. The human liver single-cell RNA-sequencing resource of Ramachandran et al. [12] is deposited in the Gene Expression Omnibus under accession GSE136103 and in Edinburgh DataShare as the processed Seurat object tissue.rdata (DS_10283_3433)The derived data supporting the reported findings are given in full in Supplementary Tables S1–S4 (Supplementary_Tables.xlsx), published with this article. The complete analysis code, the full set of derived result tables, the per-figure source data and a pinned environment specification are available from the corresponding author on reasonable request.

## Supplementary Materials

The following supporting information can be downloaded at: https://www.mdpi.com/article/10.3390/ijms27188023/s1.

## Abbreviations

AUROC	area under the receiver-operating-characteristic curve
ALD	alcohol-related liver disease
BH	Benjamini–Hochberg
CI	confidence interval
CV	cross-validation
DE	differential expression
ECM	extracellular matrix
GEO	Gene Expression Omnibus
GRN	gene regulatory network
HVG	highly variable gene
KO	knockout
LR	ligand–receptor
MASLD	metabolic dysfunction-associated steatotic liver disease
MP	mononuclear phagocyte
NAFLD	non-alcoholic fatty liver disease
OE	over-expression
PBC	primary biliary cholangitis
PCA	principal component analysis
QC	quality control
SD	standard deviation
SHAP	SHapley Additive exPlanations
scRNA-seq	single-cell RNA sequencing
TF	transcription factor
TGF-β	transforming growth factor beta
UMAP	uniform manifold approximation and projection
XAI	explainable artificial intelligence
