# Supplementary material for: Explainable Artificial Intelligence and Gene-Network Analysis Prioritise a TREM2/LGALS3/GPNMB Scar-Associated Macrophage Programme in Human Liver Cirrhosis
Source: Int J Mol Sci. 2026 Sep 9;27(18):8023. doi: 10.3390/ijms27188023 (PMC13606852; doi:10.3390/ijms27188023)
Supplement: Supplementary file 1 [file ijms-27-08023-s001.zip › Supplementary Materials.pdf]

Manuscript ijms-4422904

Explainable Artificial Intelligence and Gene-Network Analysis Prioritise a TREM2/LGALS3/GPNMB Scar-Associated Macrophage Programme in Human Liver Cirrhosis

Supplementary Figures

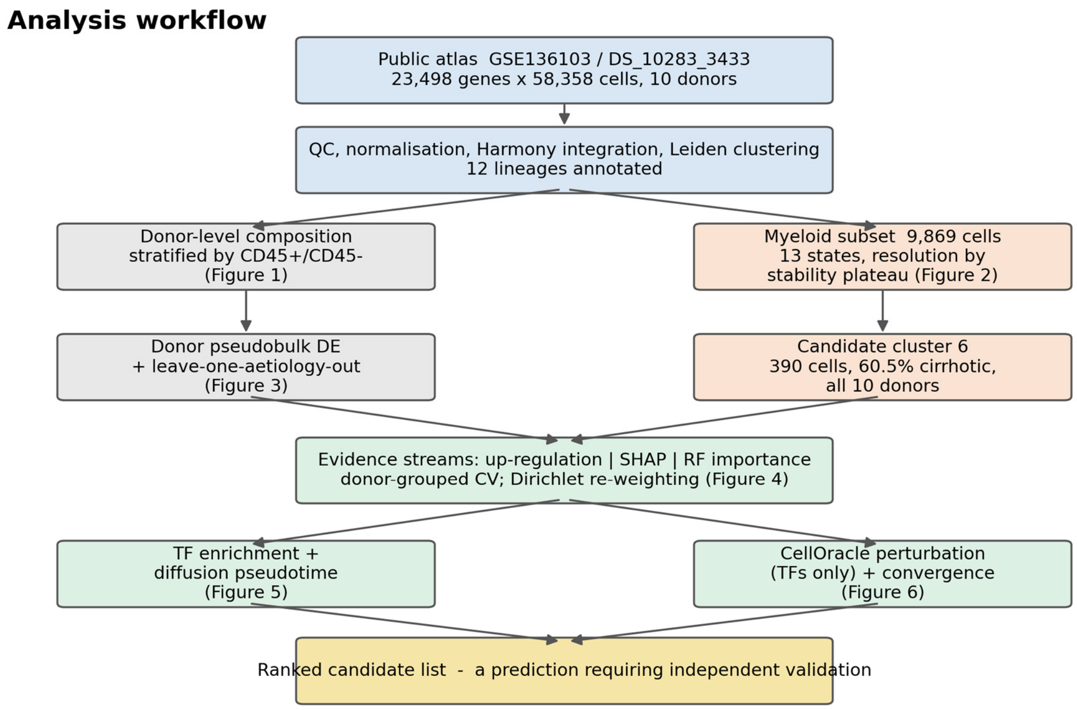

Figure S1. Analysis workflow. The complete pipeline from the public atlas to the ranked candidate list, with the figure in which each step is reported. Every step is implemented in the analysis code and regenerated end-to-end from the raw object.

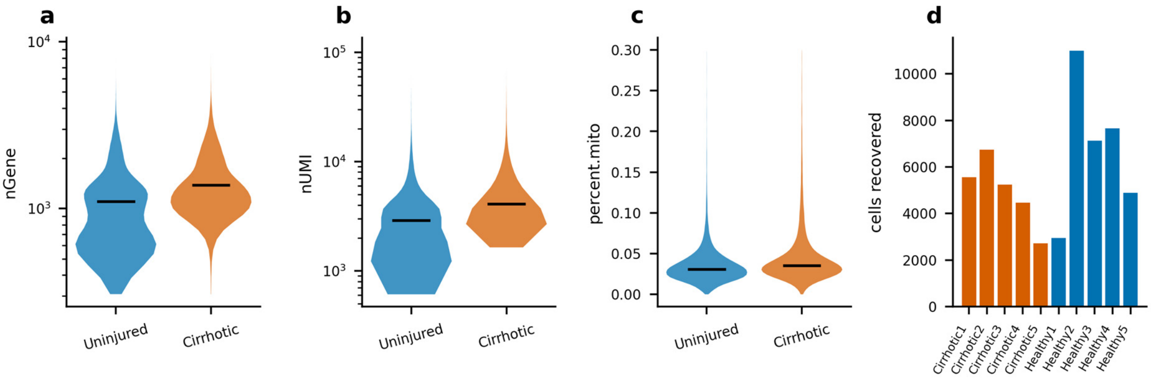

Figure S2. Quality-control metrics. (a-c) Detected genes, unique molecular identifiers and mitochondrial fraction per cell by condition; gene and UMI axes are logarithmic and horizontal bars are medians. (d) Cells recovered per donor.

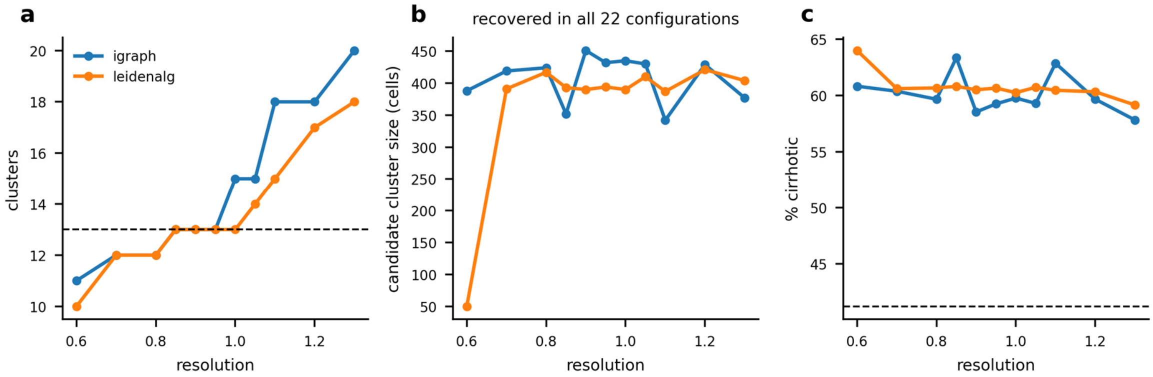

Figure S3. Clustering robustness across 22 configurations. (a) Number of clusters against Leiden resolution under two graph-partitioning implementations; the dashed line marks 13 states. (b) Size and (c) cirrhotic fraction of the candidate cluster; the dashed line in (c) is the myeloid baseline of 41.2%. A scar-programme-high cluster is recovered in every configuration tested; in 21 of the 22 it comprises 342-451 cells at 57.8-63.4% cirrhotic. The exception is the lowest resolution (0.6), which under-partitions the data and yields a 50-cell fragment.

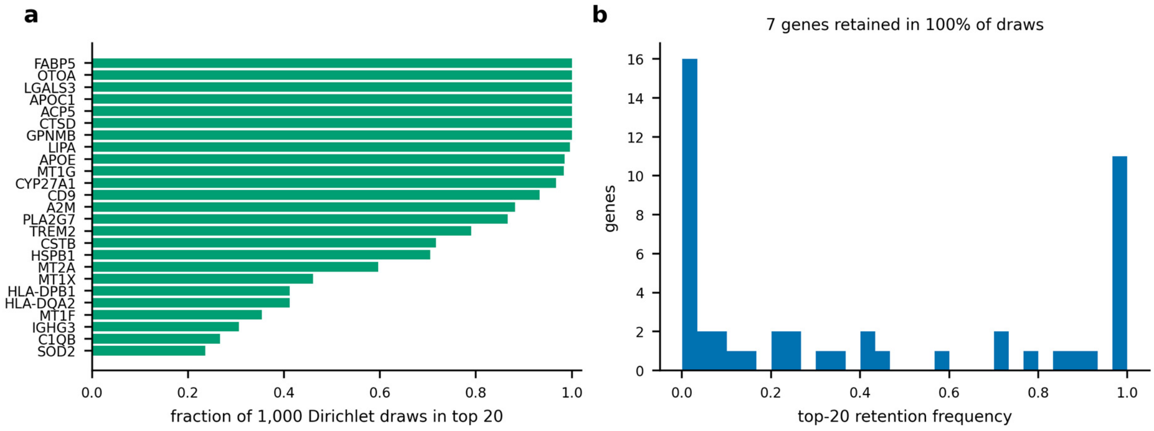

Figure S4. Sensitivity of the integrated ranking to stream weighting. (a) Fraction of 1,000 Dirichlet draws in which each gene remains in the top 20. (b) Distribution of that retention frequency across genes. Seven genes are retained in every draw.

Supplementary Tables

Table S1. Granulation tissue, fibrosis and fibroma compared. This is a contextual reference table compiled from established histopathological classifications (Gurtner et al., Nature 2008; Wynn and Ramalingam, Nat Med 2012; Sbaraglia et al., Pathologica 2021). It is NOT derived from the single-cell dataset analysed in this study, and no conclusion in the manuscript depends on it; it is provided only to disambiguate three terms that are frequently conflated. Neither granulation tissue nor fibroma is present in, or inferable from, these data. *(n = 8 rows; full data in Supplementary_Tables.xlsx, sheet 'S1_fibrosis_vs_fibroma')*

Feature	Granulation tissue	Fibrosis	Fibroma
**Nature of process**	Transient reparative response	Persistent excess matrix deposition	Benign clonal neoplasm
**Clonality**	Polyclonal	Polyclonal	Clonal
**Vascularity**	High (neovascular granulation)	Low to moderate	Low
**Principal cell**	Myofibroblast, endothelium, macrophage	Activated myofibroblast	Neoplastic fibroblast
**Matrix**	Provisional (fibronectin, collagen III)	Mature fibrillar collagen I/III	Collagenous stroma
**Reversibility**	Resolves on healing	Potentially reversible if injury removed	Does not regress; excised
**Natural history**	Replaced by scar or resolves	May progress to cirrhosis	Static or slowly enlarging mass
**Relevance here**	Not the subject of this study	The process analysed	Not the subject of this study

Table S2. Donor-level compositional statistics for every lineage in each sorted compartment. Includes bootstrap confidence intervals, Cliff's delta and the minimum difference detectable at 80%

power. No lineage is significant after Benjamini-Hochberg correction. *(n = 24 rows; full data in Supplementary_Tables.xlsx, sheet 'S2_composition_statistics')*

Table S3. Recovery of the candidate cluster across 22 resolution x implementation configurations. *(n = 22 rows; full data in Supplementary_Tables.xlsx, sheet 'S3_clustering_robustness')*

Table S4. Leave-one-aetiology-out donor-level differential expression. Programme genes and the 100 strongest genes per contrast; the complete table is available from the corresponding author on reasonable request. *(n = 327 rows; full data in Supplementary_Tables.xlsx, sheet 'S4_leave_one_aetiology_out')*
